# Hmong Older Adults’ End-of-Life Care Preferences: Physical, Psychosocial, Cultural, Religious, and Spiritual

**DOI:** 10.1093/geroni/igab046.1953

**Published:** 2021-12-17

**Authors:** Youhung Her-Xiong

**Affiliations:** William S. Middleton Memorial Veterans Hospital, Altoona, Wisconsin, United States

## Abstract

As the Hmong community continues to grow and age in the United States (US), mainstream healthcare providers may encounter Hmong older adults who prefer their cultural end-of-life (EOL) care. The challenge for these providers is to offer culturally sensitive EOL care to Hmong older adults within the realm of the Western healthcare system. One factor contributing to this challenge may be the lack of knowledge regarding Hmong older adults’ EOL care preferences. Another is Hmong EOL care is interwoven with care from domains such as culture, religion, and spirituality. The purpose of this study is to garner an understanding of the care preferences of Hmong older adults during the dying process. A qualitative study using inductive content analysis was conducted. Thirty Hmong older adults who reside in Wisconsin participated in semi-structured interviews that were audio recorded and transcribed. Data was analyzed using inductive content analysis by Elo & Kyngäs (2008). The findings revealed that participants preferred care at EOL in the domains: physical, psychosocial, cultural, religious, and spiritual. Physical care included ADL’s while psychosocial care related to communication and companionship. Cultural care included children as caregivers and decision-makers. Religious and spiritual care surrounded Animism and Christian beliefs such as soul calling and prayers. Findings also suggest Hmong older adults’ care preferences as heterogenous and holistic. The findings have implications for the Hmong community and formal care services to collaborate on how culturally sensitive care can be provided to Hmong older adults at end of life.

